# The Construction of Physics as a Quintessentially Masculine Subject: Young People’s Perceptions of Gender Issues in Access to Physics

**DOI:** 10.1007/s11199-016-0669-z

**Published:** 2016-09-06

**Authors:** Becky Francis, Louise Archer, Julie Moote, Jen DeWitt, Emily MacLeod, Lucy Yeomans

**Affiliations:** 1 Institute of Education, University College London, 20 Bedford Way, London, WC1H 0AL UK; 2 Department of Education & Professional Studies, King’s College London, Waterloo Bridge Wing, Franklin-Wilkins Building, Waterloo Road, London, SE1 9NH UK

**Keywords:** Gender equality, STEM, Education policy, Gender, Physics, Masculinity, Femininity

## Abstract

The present article investigates explanations for gendered trends in Physics and Engineering access, reporting findings from a large-scale study funded by the UK Economic and Social Research Council and drawing primarily on data from interviews with 132 15–16 year-old adolescents and their parents. Survey results in our study and elsewhere show strong gender disparities in anticipated pursuit of Physics after completion of compulsory education. In order to explore the constructions of gender and Physics underlying these trends, we focus on qualitative interview data, applying Foucaultian analysis of discourse to investigate gendered narratives underpinning adolescents’ and their parents’ articulations. This analysis reveals three key discourses at work on the topic of women’s access to Physics: (a) equality of opportunity, (b) continued gender discrimination in and around Physics, and (c) Physics as quintessentially masculine. We additionally identify five distinct narratives supporting the discourse of physics as masculine. These various discourses and narratives are interrogated, and their implications explored. We conclude that it is only by disrupting prevalent constructions of the Physical sciences as a masculine and “hard” domain will we increase the presence of women in the sector. Working with young people to analyse and deconstruct the discursive assumptions made in relation to gender and Physics, as well as further work to increase accessibility and broaden representation in Physics, may be fruitful ways to challenge these longstanding associations between Physics and masculinity.

Issues of access to, participation in, and engagement with Science, Technology, Engineering and Math (STEM) continue to preoccupy policymakers, scholarly institutions, and employers (e.g., Australian Council of Learned Academies [ACOLA] 2013; Danish EU Presidency 2012, HM Treasury 2011; U.S. President’s Council of Advisors on Science and Technology 2010). Across the Global North, there is an impetus to increase the number of people studying and working in STEM at all levels because STEM industries are deemed vital elements of the current and future economy (Confederation of British Industry 2012; Council of Canadian Academies 2015; House of Lords 2012; Landivar 2013; U.S. Chamber of Commerce Foundation 2015). Although debates remain over the number of future STEM professionals that the UK economy needs (Lowell et al. 2009; Osborne 2010; Xie and Killewald 2012), there is widespread concern from government and business about a growing gender gap in STEM skills. This gap is particularly acute in the sectors of physics and engineering (House of Lords 2012; Royal Academy of Engineering 2012). Participation rates in “core” STEM subjects (e.g., mathematics, physics) still represent a very low proportion of the overall STEM figure, with research showing that many young people do not consider continued study of these subjects as being “for me” (Archer et al. 2012; Brown et al. 2008; Hutchinson and Bentley 2011; Institute of Mechanical Engineers 2010; Larson 2014; Lewis et al. 2009; Tripney et al. 2010)*.* The situation is especially acute for Physics (Institute of Physics 2012; Smith 2010a).

As noted by organisations such as the UK’s Equal Opportunities Commission (2006) and The Campaign for Science and Engineering (CaSE; 2014, one obvious way to increase entry to Physics and other key STEM subjects is by widening participation and access to these subjects and related careers. Widening access to STEM higher study and careers comprises an equity issue for various reasons. Scientifically literate individuals are able to access well remunerated occupations (Greenwood et al. 2011). But also, many argue the importance of science literacy for civic participation because it enables citizens to understand and shape scientific developments in society (Osborne 2010). Further, Archer et al. (2012) argue that science literacy comprises a currency for social status. Drawing on Bourdieu’s (1984) theories of capital, they argue that science knowledge functions as a form of symbolic cultural capital which can facilitate agency and the re/production of privilege. In other words, science knowledge is an asset which benefits individuals in terms of their social status and access to civic debate and influence, in addition to benefitting occupational remuneration.

Furthermore, as Archer et al. (2012) argue, at present this science knowledge (“science capital”) is too unevenly spread across society. In the UK, as in many Western nations, participation in post-16 science and mathematics varies considerably by gender, ethnicity, and social class (Gorard and See 2009; Royal Society 2008; Seymour and Hewitt 1997). Women, working-class students, and those from particular minority ethnic backgrounds (e.g., Black Caribbean, Pakistani/ Bangladeshi) are under-represented in the physical sciences, engineering and mathematics at degree level (Archer et al. 2015; Elias et al. 2006; Gibb 2015; Institute of Physics 2012; Seymour and Hewitt 1997; Smith 2010a, b).

The last 40 years have seen notable improvements in gender equity within science in many Western nations (e.g., American Association of University Women 2010). However, entrenched gender inequalities still persist: Gender inequalities remain both in terms of students’ *perceptions* of science and in their patterns of *participation* in post-16 physical sciences and engineering. Middle-class, White, and South/East Asian heritage young men are most likely to study the physical sciences and engineering at degree level, a pattern which has not changed for many years (Smith 2011). These gendered patterns persist despite scant gender differentiation in attainment in school science (Haworth et al. 2008; Royal Society 2008; Smith 2011) and mathematics (Boaler and Sengupta-Irving 2006). As Seymour and Hewitt (1997) showed, women and minority ethnic students are more likely to drop out of STEM degree courses despite parity in course entry levels (Seymour and Hewitt 1997). These patterns endure in spite of innumerable programmes and initiatives to encourage female participation in science study and careers over the last 40 years (Danish EU Presidency 2012; Royal Society 2008; Smith 2010a; Smith and Gorard 2011).

## Inequality in the Physical Sciences

So why do these patterns of entry to the physical sciences and engineering persist? As we have seen, prior attainment is not an explanation for gendered patterns in uptake. And although differential prior attainment may contribute in part in the case of working class students and those from particular minority ethnic backgrounds, other factors have been shown to play a role in lower progression rates. For example, Strand’s (2012) analysis of UK longitudinal data found that minority ethnic (but particularly Black Caribbean) students are less likely to be entered into higher tier examinations than are White students, even after controlling for prior attainment. Differential teacher expectations have also been demonstrated to impact with regard to gender: Carlone’s (2003) research shows how teachers tend to attribute girls’ achievement in Physics to their “hard work” but regard boys as “naturally bright” at Physics, even when they attain less highly than their female peers. Further, Mujtaba and Reiss (2013) found that young women receive less encouragement from teachers, family, and friends to study Physics post-16 in comparison with young men.

Students who are traditionally under-represented in post-16 physical sciences and mathematics (notably young women, working-class, and certain minority ethnic young men) also tend to articulate less confidence in their own abilities and are less likely to identify themselves as being “good” at science and/or mathematics, irrespective of their actual abilities and attainment (Cheryan et al. 2011; Mendick 2005; Mujtaba and Reiss 2013). Feminist social constructionist research has explored these trends in relation to the gendered construction of science and scientists. This body of work argues that science is socially constructed as a high status, masculine domain that is appropriate for, and populated by, middle class men. As such, young women, and young men from working class and certain minority ethnic backgrounds, are discouraged from the pursuit of science, directly or indirectly.

It has been shown that young people tend to associate most science careers with masculinity (Archer et al. 2012), with children perceiving science as being “for boys” (Calabrese Barton and Tan 2009; Caleon and Subramaniam 2008; Carlone 2003; Farenga and Joyce 1999; Fennema and Peterson 1985; Francis 2000; Mendick 2005). Respondents also perceive STEM occupations to be individualistic, that is, not involving working with or helping others, which in turn has been shown to deter women (Diekman et al. 2011). The construction of Physics (especially) as masculine (Gonsalves 2014) has been shown to impede many young women’s identification with Physics by challenging their construction of femininity (Archer et al. 2016a). Evidence suggests that young people continue to regard science and mathematics as White, male, middle-class pursuits—with scientists and mathematicians tending to be imagined as White middle-class men (Archer et al. 2015; Cheryan et al. 2011, 2013; Mendick 2005; Wong 2012)—which in some areas of the sciences, and particularly at senior levels, may be the case.

Moreover, as feminist researchers such as Harding (1991, 1982) and Walkerdine (1988, 1989) have asserted, STEM disciplines are constructed upon, and perpetuate, longstanding epistemological, enlightenment constructions of reason, intellect, and competition that are, in turn, historically associated with masculinity. Several studies have explored how such associations between STEM and masculinity impede girls/women’s identification with STEM (Walkerdine 1990) and/or necessitate those engaged with STEM to adopt particular strategies to bridge this identification challenge. For example, Pronin et al. (2004) found that women invested in maths adopted “bifurcation”—disassociating themselves from feminine stereotypes in relation to math. Similarly, Archer et al. (2016a) found that young women who identified with Physics tended to describe themselves as unfeminine. Critiquing the masculinist discourses that maintain such associations between STEM and masculinity, and which therefore exclude femininity, Walkerdine (1990) and others have adopted poststructuralist theoretical lenses to deconstruct the gendered discourses that perpetuate these productions of STEM. It is these conceptual understandings of the social production of gender difference and of science as a masculine domain that we build upon in our approach to our research and data analysis.

In summary then, the existing literature highlights that understanding the factors which deter young people from pursuing routes into the physical sciences and engineering remains a key priority, both for Governments focused on economic productivity and in terms of equity, civic participation, equality of opportunity, and social justice. Especially, questions remain concerning the ongoing lack of access to Physics for women (and for men from working class and some minority ethnic backgrounds), as well as what might help to reverse this pattern. Research has established that STEM subjects, and Physics in particular, continue to be constructed as masculine, precipitating various practices that deter girls from pursuing these subjects for higher study. However, there has been less attention to how individuals explain these trends or the discourses underpinning such explanations.

## Theoretical Perspective

Building on the feminist, social constructionist perspectives outlined previously, our theoretical approach comprises a “deep” social constructionism informed by Foucaultian poststructuralism (Fraser and Nicholson 1990). We understand gender to be socially constructed as *binarised* (i.e., bodies constructed as divided into male and female, with binarised characteristics ascribed to each, and these resulting constructions of masculinity and femininity as relational to each other). This dualistic construction is maintained by gender *discourses* (Butler 1993; Davies 1989), with discourses being language patterns and practices that constitute the objects of which they speak and that bear power in their ability to produce objects and subjects in different ways (Foucault 1980). Butler (1993) has drawn on Foucaultian theory to analyse how gender and sexuality discourses delineate the normal and abnormal, acceptable and unacceptable. She explains how, in this sense, binarised discourses of gender and sexuality produce some subjects, and their bodies and/or behaviours, as natural (“intelligible”) and others as unnatural and abnormal, that is, as “unintelligible.” She elaborates how these discourses order and police gender/sexuality productions and render untenable those productions which do not conform to binarised norms. Such “unintelligible” bodies disrupt the gender/sexuality order, and therefore those subjects expressing them are discursively positioned as “impossible subjects” (Butler 1993). Such positioning placed these “impossible selves” as at risk of social discipline and punishment. Foucaultian analysis of discourse (Burman and Parker 1993; Foucault 1980) can be applied to make visible and interrogate these discourses and thereby to potentially deconstruct the dualistic constructions that they support.

We also draw on Bakhtin’s (1981) constructs of monoglossia and heteroglossia. Analysing language, Bakhtin uses the term *monoglossia* to refer to dominant forms of language, representing the world-view/interests of dominant social groups, which are positioned or imposed as unitary and total. However, for Bakhtin, language is never static or fixed; rather it is diverse and inherently dialogic. Different meanings and readings constantly jostle in assertions or subversions as subjects use language in different ways. Hence whereas at the macro-linguistic level there may appear to be stability (monoglossia), at the micro level there is plasticity, contradiction, and resistance, that is, *heteroglossia*. Francis (2012) has applied these conceptual tools to the construct of gender itself. She argues that the dualistic account of gender (with its animation of the subject as masculine, and denigration of the feminine as Other, as an integral element of the dualism) is monoglossic: It authors itself as “real,” “natural,” and “total.” Yet it is infused at every level with heteroglossic contradiction and potential disruption, in both the theory and performance of gender (Francis 2010, 2012). Application of these theoretical tools can help to explore and explain the simultaneous hegemony and fluidity of gender constructions.

## The Present Study

Building on the existing literature, we seek in the present article to explore respondents’ constructions of gender and access to the physical sciences, with particular attention to their explanations for gender inequality in this area. A key research question for the wider study was the extent to which our data reflect prior research findings showing gender inequalities in proportions of students’ intending to pursue Physics for further study after compulsory schooling. Distinctively, we also sought to ask young people and their parents directly for their opinions on why fewer women pursue the Physical sciences in order to explore the different discourses produced in response. This approach is intended to enable identification of the range and nature of discourses that are applied on the topic of gender inequalities in access to Physics, as well as the ways in which these discourses work to construct the Physics discipline, and gendered subjects, in different ways.

## Method

### Overview

The data we analysed were generated by the Economic and Social Research Council-funded “Young People’s Science and Career Aspirations age 14-19” (ASPIRES 2) project. The longitudinal study, and its predecessor “ASPIRES” study, have been tracking and exploring children’s science and career aspirations from ages 10–19. Methods include a quantitative online survey of the cohort and repeated interviews with a sub-sample of students and their parents. In the present project phase, these methods have been applied when students are age 15/16 years-old (Year 11). Our paper draws primarily on the qualitative interview data generated from the interviews with young people and their parents in the present project phase. The present study subscribes to the ethical standards of the British Educational Research Association, and it has been appraised and approved by the ethics committee of King’s College London.

The present paper reports only briefly on elements of the quantitative survey findings. Nevertheless, given that some findings from the survey are alluded to and precipitate the qualitative investigation, we briefly outline the survey method here. The survey collected a range of demographic data, and it covered topics such as aspirations (including a focus on science); subject preferences; science participation in and outside school; self concept in relation to, and perceptions of, Physics, Biology, and Chemistry; parental and peer attitudes; and careers education. All items were validated (see Archer et al. 2016b). The survey was sent to a nationally representative sample of 340 schools in England (296 state schools and 44 independent), and it was completed by 13,421 students. Schools were invited to arrange for one or more mixed attainment classes, science sets, or tutor groups of Year 11 pupils (aged 15/16 years) to complete a 30-min online questionnaire in Autumn 2014. Schools were also encouraged to invite additional classes (e.g., a spread of top, middle, and bottom sets, or entire cohorts) to participate in order to receive a more comprehensive picture of students’ attitudes toward science and their career aspirations.

### Participants

Of the 13,421 students who completed the survey, 6266 (46.7 %) were male and 7155 (53.3 %) were female; 6561 (49 %) were categorised as social class 1 (most affluent, professional and higher managerial), 3764 (28 %) as social class 2 (lower middle class; skilled occupations), 1498 (11 %) as social class 3 (upper working class; semi-skilled or unskilled), 761 (6 %) as social class 4 (manual working class), and 819 (6 %) were uncategorised. We gathered data on our survey relating to parental occupation, which was used in analyses as a proxy for socio-economic classification (recognising that SES and the related notion of social class are complex and contestable concepts). Pupils were asked about their mother’s and father’s (or caregiver’s) occupations, and categorisations were assigned based on the highest recorded occupation. Due to the complexities of asking children about their parents’ occupations, a simplified categorisation task was developed and used (as opposed to using the full ONS SEC categorisation system, which proved to be too cumbersome and time-consuming during piloting). Respondents self-reported as White (10,181, 75.9 %), Asian (1306, 9.7 %), Middle Eastern (122, 0.9 %), Black (503, 3.7 %), Chinese/East Asian (205, 1.5 %), Mixed/Other (648, 4.8 %), and 456 (3.4 %) of students preferred not to answer.

The present article primarily draws on qualitative data from 132 interviews (in the current project phase) with 70 15/16 year-old students (30 young men, 40 young women) and 66 of their parents (16 men, 50 women). All these respondents had been previously tracked since students were aged 10/11. The recruited interview respondents came from a broad range of socioeconomic classes and ethnic backgrounds: White British (15 boys and 26 girls), White European (2 boys, 3 girls), British Asian (3 boys, 1 girl), Asian (1 boy, 2 girls) and Black African/Caribbean (3 boys and 3 girls), Mixed (6 boys, 5 girls). When drawing data for our article, we recorded each respondent’s pseudonym (which they chose themselves when first interviewed at age 10/11), gender, ethnic heritage, and social class category. The latter categorisation is based on information gained from interviews such as parental occupations (using the NS-SEC categorisations), housing tenure, and parental educational backgrounds. Categorisations run from 1 to 4, where 1 represents professional and/or highly educated parental backgrounds and 4 represents minimally educated/manual unskilled parental occupations.

Interview participants were originally recruited from 11 schools in England (one in the Midlands, two in the eastern region, two in the south east, four in London, and one in the south). These schools were sampled from the 279 schools that responded to the Phase 1 survey as part of the wider study (see Archer et al. 2012 for details). A sampling frame was developed to represent six target categories of school (“multiethnic urban/inner city schools,” “working-class suburban,” “predominantly White, middle-class suburban schools,” and “independent single sex”) to ensure a range of school contexts and populations. The prospective schools for interviews were purposively sampled using these target categories. Over the course of the project, students were tracked as they moved through to elementary and secondary school.

### Interviews

Interviews lasting approximately 45 min were conducted by four of the paper authors, with the majority of the interviews conducted by the third author. Of the interviewers, three (BF, LA, JM) are White middle-class women (with English and Canadian national backgrounds), and one (LY) is a White woman Ph.D. student of working class heritage. We see the interview encounter as contextually situated and are interested in the discourses precipitated therein (Burman and Parker 1993). The interviews took place in a private room at school or in an alternative private location chosen by the students and their parents (e.g., the home, work office, via telephone). Two topic guides were developed and piloted with parents and students covering areas including aspirations (and sources of these aspirations), interests in school and outside school, what they like/dislike about school, attitudes toward and engagement in school science, and broader perceptions and engagement with STEM subjects. Parental interviews included additional focus on family context including perceptions and experience of the child’s schooling, involvement in education and careers education provision, as well as the child’s interests and aspirations. We also asked students and parents for their reflections and opinions on the under-representation of women in Physics, aiming to analyse the various discourses produced and narratives underpinning them. In this section of the interview we also asked respondents about women’s under-representation in Engineering, if time allowed, given this is also a discipline within the Physical sciences wherein women’s under-representation is well documented (Perkins 2013; Royal Academy of Engineering 2012). It is this latter section of the interview questions focusing on the under-representation of women in the Physical Sciences that form the focus of analysis in the present article.

All interviews were fully transcribed and thematically organised via NVivo. Data were then subject to Foucaultian discourse analysis following the methods advocated by Burman and Parker (1993) and elaborated regarding the distinction between discourses and narratives by Francis (1999). As such, we use “discourses” to comprise the broad content and active construction of a topic that constitutes subjects and objects in particular ways (also commonly referred to as a “grand narrative” or “meta narrative”) and “narratives” to capture the various, more specific sub-discourses that both articulate and support the wider discourse (Francis 1999). The analysis of emerging discourses was assessed against the original texts by Authors 2 and 4. An additional theoretical layer is applied to the analysis via application of the concepts of monoglossia and heteroglossia (Bakhtin 1981), and of gender monoglossia and gender heteroglossia (Francis 2012), in order to explain the dominance and diversity of particular gender constructions.

## Results

### Plans to Study Physics

In England, compulsory schooling is concluded by GCSE examinations at the end of Year 11 at age 16. “A Level” qualifications represent the academic study route pursued by young people in Years 12 and 13, when they are 16–18 years-old, and they form the main entry route to university Higher Education. Our survey findings show that, of those 9216 students in our study planning to continue with full-time post-compulsory education, 23 % (*n* = 2143) planned to pursue Physics at A Level. This is a significantly higher proportion than is reflected in national statistics for the proportion of students pursuing Physics A Level (8 %: see Questions for Governors 2014a), likely reflecting our respondents’ aspirations rather than actual registration for the course and our sample’s apparent inclusion of a disproportionate number of pupils pursuing the Triple Science route (the curriculum route involving separate science qualifications for Physics, Biology, and Chemistry, which is usually open only to higher attaining students). Nevertheless, of the 2143 student respondents planning to pursue Physics, only a third (35 %, *n* = 756) were female (compared to 21 % nationally; Questions for Governors 2014b). Hence, even within our somewhat “science-focused” sample, the trend for male domination of Physics is strongly evidenced. This returns us to the longstanding question as to why more men than women pursue Physics study and careers.

Our quantitative findings also demonstrate that the pursuit of Physics is strongly related to social class, with almost all students planning to pursue Physics A Level coming from the highest social class 1 and 2 categories (85 %, *n* = 1820). Furthermore, students from certain minority ethnic groups (notably South Asian) are disproportionately represented (13.2 %, *n* = 282 compared to 9.7 %, *n* = 1306 for the whole sample). These different variables clearly intersect with gender in access to Physics. The relationship between gender and social class concerning identification with Physics (or otherwise) is explored further in Archer et al. (2016a).

### Barriers to Pursuing Physics and Engineering

Although social class and ethnicity clearly pattern access to Physics in addition to gender, we did not find distinctive social class and ethnicity patterns in relation to the construction of Physics as gendered. Rather, students from different social and ethnic backgrounds appeared equally likely to subscribe to, or to reject, gendered constructions of Physics. Respondents from different ethnic and social class backgrounds also appeared no more or less likely to mobilise different discourses in their explanations. (Albeit numbers for different groups were small, so this finding should be treated with caution.) This diversity is illustrated by the quotes from the qualitative data we cite.

In contrast, some gendered trends in responses were evident. Asked “Do you think there is anything that is putting women off pursuing careers in Physics?,” it was interesting to see a gender discrepancy in interview responses: less than a quarter of young men (*n* = 7 of 30) felt there might be things that put women off pursuing careers in Physics, with more male adolescents (*n* = 12) either simply answering “no there isn’t,” or offering equal opportunities and individual meritocracy discourses to argue that there is nothing deterring women. This was in comparison to more than half of young women (*n* = 22 of 40) who felt there were things that put women off—with gender discrimination and stereotyping as notable explanations among these accounts. Only 10 girls said there was nothing deterring women from pursuing Physics careers (the remaining 4 girls, and 6 boys, gave ambiguous responses or said they did not know).

However, as Table 1 shows, young men were more likely to change their minds when probed about the case of Engineering. Although not all students were probed on this point (48 were asked the question, 22 not), in contrast with their responses concerning Physics careers, very few young men (*n* = 3) said that there is nothing putting women off pursuing engineering careers, and 12 said there were (various) reasons, with four additional young men providing ambiguous answers or “don’t know” responses. Sixteen young women agreed that there are barriers to women pursuing engineering careers, with only three saying there is nothing deterring women (a further three young women either did not know or provided ambiguous responses). These responses appear to chime with the particularly gendered profile of engineering (Perkins 2013).

**Table 1:** Question responses, descriptions, examples, and frequencies

		Comments from students				Comments from parents				Total participants (*N* = 136)
Response type category	Description	Example				Example				
			Male (*n* = 30)	Female (*n* = 40)	Total (*n* = 70)		Male (*n* = 16)	Female (*n* = 50)	Total (*n* = 66)	
Physics										
Women are put off Physics	Believes that something is putting women off careers in Physics	“I think women think it’s more of a men’s like topic. I do think that... Physics and Engineering is more for men, which I think puts off women because I don’t think they’d be able to have the capability to do it” (Carol, White European girl, social class 3)	7	22	29	“I think sometimes as well once they get there it’s such a male dominated field that they have to work so much harder to be recognised” (Sandra, parent of Danielle, White British female, social class 3)	5	24	29	58
Women are not put off Physics	Believes that nothing is putting women off careers in Physics	“I think there probably would have been in the past. But now a lot more women are taking part in pretty much anything now. I don’t think there’s that divide anymore” (Lucy, White British girl, social class 4)	12	10	22	“If you’re a science-y person I think it’s fine, but you know it all depends, you know, on the person to be honest... And so you know you’ve got to be good at science stuff or Physics stuff to want to be doing it anyway” (Stella, parent of Josh, White British female, social class 3/2)	4	8	12	34
Unsure if women are put off Physics	Neither agrees nor disagrees that women are put off Physics	“I’m not sure... Um, I think the thing that’s putting them off mostly is probably the fact that it’s already like a male dominated field, but like other than that like quite a few of my friends who are girls are doing sort of double Maths and doing Science A levels, so…” (Robert M, White British boy, social class 1)	9	4	13	“It’s not very often you can see the girls or women in that profession, because these are more boys involved in these ones – especially for engineers. But maybe the future will change that thing, I don’t know why” (Grigor, parent of Victoria2, White European male, social class 4)	3	9	12	25
People in general (male and female) are put off Physics	Believes that something is putting both men and women off Physics	“I would say that for both it’s just the thing that it’s a lot of maths. And if you don’t enjoy maths then you’re not going to enjoy it, so people are kind of steering away from it” (Tom4, British Asian, social class unknown)	15	20	35	“I would say with Physics... what puts people off is it’s seen as quite hard. So I think that’s probably a bigger factor than whether it’s the right thing for a girl to do or for a boy to do” (Janet, parent of Gus, White British female, social class 1)	2	7	9	44
People in general (male and female) are not put off Physics	Believes that nothing is putting both men and women off Physics	“No I wouldn’t say so. Just, it’s whatever you’re interested in. Like I’m interested in Maths and that, but other kids might be interested in like business or something” (Hedgehog, White British boy, social class 4)	7	4	11	“No I don’t think so to be honest, I think it’s just they’ve got to have the interest and the want to do it” (Carolyn, parent of Brittney & Celina2, White British female, social class 3)	1	3	4	15
Unsure if people in general (male and female) are put off Physics	Neither agrees nor disagrees that people in general (men & women) are put off Physics	“I don’t know. I’m not, I’m not a massive fan of Physics... I find it hard to understand why people would want to study it. I do, I do sometimes wonder why people want to study it, but it’s their choice. Like they might really enjoy it” (Louise, White British girl, social class unknown)	4	6	10	“I can’t think of anything… No I can’t think of anything really that would put them off” (Dennis, parent of Bill, White British male, social class 1)	2	7	9	19
Feminine girls do not want to study Physics	Believes that ‘girly’ girls are less likely to pursue Physics than their peers	“Girls that are very girly, they wouldn’t exactly want to get into that. Maybe they wouldn’t want to get into all hard work and stuff like that... A girly girl isn’t going to be into that kind of stuff – it’s very unlikely. They’d probably [be] into maybe different things that are more suited towards them creative things maybe” (Kaka, British Asian boy, social class 2)	18	19	37	“‘Cos if they’re a girly girl I can’t see them like wandering around in a lab in white coat – they’d want to be out where they’re more with the public and dolled to the nines doing something that’s a bit more like, you know, with the public. Do you know what I mean? Even if it’s in shop work or an office, something where they can, you know, wear their full make-up... as opposed to in a lab and a white coat” (Robyn, parent of Charlie, White British female, social class 2/3)	7	24	31	68
Feminine girls are not less likely to want to study Physics	Believes that ‘girly’ girls are no less likely to pursue Physics than their peers	“I think everyone just has different interests and they just mix up, I don’t think it’s like if you’re really Physics-y you have to be like a tomboy” (Luna, White British girl, social class 3)	8	14	22	“I don’t know that that necessarily they’re less likely to want to pursue Physics, it’s just they probably have an image that other people perceive is that they don’t want to be scientists – just what they look like doesn’t make them not interested in Science” (Ella, parent of David, White British female, social class 2/3)	8	15	23	45
Engineering										
		Comments from students				Comments from parents				
			Male (*n* = 19, of 30)	Female (*n* = 29, of 40)	Total asked (*n* = 48, of 70)		Male (*n* = 14, of 16)	Female (*n* = 35, of 50)	Total asked (*n* = 49, of 66)	Total participants (*N* = 97, of136)
Women are put off Engineering	Believes that something is putting women off careers in Engineering	“Like female engineers, everyone needs them, um, but like I think there’s still that slight stigmatism of oh that’s a man’s job and I think that’s probably why some girls are a bit like mm, no” (Thalia, White British girl, social class 2)	12	16	28	“There aren’t any role models are there really... I haven’t come across any role models that girls would want to aspire to in Engineering” (Ella, parent of David, White British female, social class 2/3)	5	14	19	47
Women are not put off Engineering	Believes that nothing is putting women off careers in Engineering	“I think a lot of people in my school were more inspired to do Physics because they wanted to be engineers and things. ‘Cos I think a few years ago Physics in our school, not many people took it for A Level – which I was quite surprised at, cos that’s when I really enjoyed it and I wanted to take it for A Level. But I think nowadays more people … more women want to take it because they want to go into Engineering or Physics related jobs” (Mitchy, White British girl, social class 2/3)	3	3	6	“I think there’s enough ladies out on work sites doing engineering to put that into its place now. I think women have worked an amazing pathway forward and taken on roles that were originally traditionally male” (Lottie, parent of Cheeky Monkey, White British female, social class 3)	2	7	9	15
Unsure if women are put off Engineering	Neither agrees nor disagrees that women are put off engineering	“Other than the fact that from a very young age women don’t see themselves as doing that. I don’t think there should be anything to put women off from it” (Lucy, White British girl, social class 4)	4	3	7	“Well I don’t know, cos you know the adverts have all got a girl in it somewhere, I don’t know what it is. Maybe it’s ‘cos you think about it as machinery and it still has quite a manual side to it. I don’t know, I really don’t know” (Jane1, parent of Chloe, White British female, social class 2)	1	4	5	12

### Emerging Discourses and Narratives

Table 2 sets out the three key discourses identified in discussion of this issue, and the five different narratives identified as underpinning the third discourse (that of Physics as “quintessentially masculine”). Table 2 also provides details of the number of student and parent respondents using the various discourses and their gender.

**Table 2:** Discourses and narratives describing physics as quintessentially masculine, coding, examples, and frequencies among students and parents

Discourse/Narrative	Coding definition	Example	Comments from students			Comments from parents			Total participants (*N* = 136)
			Male (*n* = 30)	Female (*n* = 40)	Total (*n* = 70)	Male (*n* = 16)	Female (*n* = 50)	Total (*n* = 66)	
Discourses on the topic of women’s access to physics									
Meritocratic equality of opportunity in Physics	Articulations positioning opportunities as equal for men and women in physics	“If they want to do something then they should be able to do it, so and I think the world’s changed now, so there should be opportunities not like it was before when it was a man’s job to do that.” (Jane2, mother of Dave, White English, F, social class 3/4)	7	10	17	4	19	23	40
Gender discrimination still operates in and around Physics	Quotes articulating the continuation of gender discrimination in physics	“I think sometimes as well once they get there it’s such a male dominated field that they have to work so much harder to be recognised.” (Sandra, mother of Danielle, White English, F, social class 3).	9	20	29	5	22	27	56
Physics (and engineering) as quintessentially masculine	Articulations positioning physics and Engineering as quintessentially masculine	“Physics itself has always been seen as a more sort of a man’s job” Victor2, White British, M, social class 3)	55	84	139	16	61	77	97
Narratives supporting the discourse of physics as quintessentially masculine									
Certain subjects are gender stereotyped as masculine or feminine (and hence as appropriate for different genders)	Articulations positioning that certain subjects are gender stereotyped as masculine or feminine	“I feel like in society it’s more acceptable for women to do jobs like being a nurse or studying medicine.” (Demi, white British, F, social class 2/3)	18	21	39	3	9	12	51
Men and women are naturally different and drawn to different subjects accordingly	Articulations positioning men and women as naturally different and thus drawn to different subjects	“I think maths is masculine because it sounds weird, but women will argue and men will say their point and leave it and maths only has one answer. Whereas english is a debate. That’s why I think english is a woman’s subject and maths is a man’s.” (Danielle, white British, F, social class 3)	9	16	25	3	13	16	41
Femininity is antithetical to (masculine) manual work	Articulations positioning femininity as antithetical to (masculine) manual work	“Like isn’t that [engineering] more manual? So there’d probably be more men in there just for the sake if it being manual labour.” (Alan, mixed, white/black caribbean, M, social class 3)	13	12	25	7	13	20	45
Femininity is superficial	Articulations positioning femininity as superficial	“Um, women like want to keep their nails clean and stuff like that and engineering if you get hands on in engineering then it’s going to, you’re going to get dirty and stuff like that and some women they’re quite like pathetic in the way they act and they’re like ‘ooh, no I don’t want to touch that’ and stuff like that.” (ghost, white, M, social class 3)	10	17	27	3	12	15	42
Cleverness is masculine, and Physics is a clever/difficult subject	Articulations positioning cleverness as masculine, and physics as a clever or difficult subject	“I think it’s because physics has always been seen as this intelligent and really hard. . and you know you have to be so clever to understand it that only the male side think that they’re fit enough to do it, and not the ladies.” (Mienie, South Asian, F, social class 2)	4	13	17	0	8	8	25

#### The Discourse of Meritocratic Equality

Of the 22 (31 %) students and 12 (18 %) parents who claimed there is nothing deterring women from careers in Physics, those who elaborated their response tended to draw on narratives of individualism and meritocracy identified as highly prevalent in other studies (Francis et al. 2013). As Victoria2 (White Bulgarian, F, social class 4) explains: “I wouldn’t think that anything is putting women off specifically because if a woman wanted to study Physics then it would be her choice.” Likewise, Gemma (Black Seychellois, F, social class 3) supplants the structural implication of the question with a narrative of individuality and agency: “No not really, it depends on their interest and stuff.” CheekyMonkey (White, M, social class 3) exemplifies how the discourse of meritocracy interweaves this narrative of “individual” ability or choice by asserting that nothing precludes young women pursuing Physics: “I think they’re [individuals] just going to just work for it.”

These accounts rest on well-analysed and closely entwined discourses of (a) neoliberal individual agency (Francis et al. 2013; Rose 1999), a discourse which interpolates individuals as agentic authors of their own outcomes depending on ability, entrepreneurship, and/or diligence (Bauman 2005) and (b) equality of opportunity, which positions gender discrimination as a thing of the past (Francis et al. 2013; Hey 2005; Volman and Ten Dam 1998). The latter was also overtly articulated in the data: “Um … I think there probably would have been in the past. But now a lot more women are taking part in pretty much anything now. I don’t think there’s that divide anymore” (Lucy, White British, F, social class 1) (see also Jane2, quoted in Table 2).

As Francis et al. (2013) argue, it is important that this widely reported, monoglossic account of opportunities as equal according to gender be acknowledged by sociological researchers and that discursive investments in individual agency be appreciated as well as critiqued. They reflect rapid social shifts in recent decades, including the impact of feminism. Nevertheless, it remains a concern that the evidence (in this case, regarding women’s access to Physics careers) belies the discourse and the risk that these individualistic narratives which consign discrimination to history thus “responsibilise” (Rose 1999) young women for their “incorrect choices” and lack of access. For example, several respondents position access to Physics as coming down to individual attributes such as “motivation” and “willpower”:

I don’t really think there’s like a thing where it puts off like females, but maybe they’re intimidated perhaps like maybe of like men in Science and stuff like that. But I don’t really think … as long as they have the motivation it shouldn’t really be a problem. (Colin, Sri Lankan, M, social class 3)

Um, to be honest at the moment no, because like girl power has got really strong recently and I think it’s more girls and women are trying to put their names and faces into things that predominantly men are meant to like be good at, so I think it’s actually more of like a willpower to go into things like that (Georgie, White British, F, social class 1)

I think it’s just themselves and the confidence to be honest—I think it’s all to do with confidence. I don’t believe that it’s a man’s world and all that nonsense anymore, I think that’s well and truly died out—women are more than capable to learn what they want to learn. (Tasha, mother of Alan, Mixed Carribean/White, F, social class 3)

Hence respondents’ rejection of the idea that women are deterred from pursuit of Physics tended to rest on discourses of equality of opportunity and meritocracy. These discourses reject and deny gender discrimination, with the potential effect of positioning women as individually responsible for their lack of access to Physics.

However, as we observed previously, a third of young men and nearly two-thirds of young women asserted that there are impediments to women’s pursuit of Physics careers (and a greater portion of young men, and half of young women, felt that there are issues that deter women from engineering). These respondents provided a variety of explanations, drawing on two key discourses (and a variety of narratives articulating these): (a) continuing gender discrimination in and around Physics and (b) Physics (and Engineering) as quintessentially masculine. An additional recurrent theme was the disproportionate representation and domination of Physics by men as an explanation for women’s lack of participation. We explore each, with additional attention to this latter theme of lack of women’s representation in Physics: this theme could express both discourses, but it was largely articulated in critiques of sexism and discrimination and was notably recurrent in participants’ responses (it was used by 61 of the 132 respondents).

#### The Discourse of Gender Discrimination

As Table 2 shows, in keeping with their stronger articulation of discrimination in the Physical sciences, this discourse was used especially frequently by young women and mothers (50 % of the 40 young women used this discourse, as did 44 % of the 50 mothers, compared to just under a third of young men and fathers). Some participants maintained that gender inequality and discrimination in Physics has not yet been overcome, or is taking time to shift—what Blackbird (Father of Finch, White British, M, social class 2/3) refers to as “a historic and cultural hangover.” Poppy (White British, F, social class 1) likewise asserts that Physics remains “male dominated.” She goes on to elaborate the gender discriminatory and essentialist perceptions articulated by one of her teachers:

Like today in Chemistry … we have a different teacher to normal because the other one’s off … and my friend said that she wanted to do higher level Maths. And apparently that is really really hard to get a 7 in [“7” refers to a high expected “level” in the English National Curriculum, applied until officially abandoned by the Government in academic year 2015/16]. And the teacher said, (she’s a girl), she said “Oh I think you have to have a boy brain to do that” … *Really?*

The whole class was like “*What?*” (laughs) “You don’t say that at this school.” And like my friend now really just wants to do it because … to prove her wrong. We don’t really understand what she was saying by a “boy brain,” She said “Oh you get boys that tend to be really geeky and good at Maths” but we were like “Well you get girls like that too.” She’s a Chemistry teacher!

Indeed, several respondents had cautionary tales of sexism that female novices had experienced in seeking to access Physics and Engineering. For example, referring to the “great shame” that women are under-represented in Physics careers, Harris (father of Emma, English/Belgian, M, social class 1) recounts that one of his daughter’s friends “had a really rough time doing the Sciences (inaudible) at a good university and really struggled—she was the only girl on the course.” Likewise, Kate (White British, F, social class 1) explains that her brother’s girlfriend is studying Engineering at university and needed to undertake a year’s study in industry as part of the course:

She went … I’ve forgotten what company it was … but in the interview they asked her how will you cope as a woman in this male company. And she was just like “What?” And then they pretended that they hadn’t asked her like such a sexist question.

Kate’s conclusion illustrates the discursive impact of such ongoing discriminatory practices on other potential female applicants:

So I think you would face issues, but probably [...] So I don’t think it *stops* you doing anything – I think it just puts pressure on you not to. I think there’s probably any job you could do in Engineering as a girl, but you’re less likely to probably.

These “cautionary tales” of sexism from teachers and gatekeepers are especially worrying given the findings about the strong impact of teacher and other “expert” expectations and their communication on young women’ self-confidence and pursuit or otherwise of Physics (Seymour and Hewitt 1997; Spears Brown and Leaper 2010; Reiss et al. 2011).

Other respondents saw additional off-putting consequences to the male domination of the field: “I think that it is a male environment, so I don’t know. I think it would be harder for a woman. [...] I think it, she would feel that she has to be extra, you know work extra hard and prove herself more all the time to compete with a male” (Patsy, mother of Indiana, White British, F, social class 3; see also Sandra, Table 2). (It is worth noting that such perceptions of women’s experiences in Physics are borne out by research studies such as Danielsson 2012 and Ong 2005.) Hence these respondents articulated this discourse of gender discrimination still operating in and around Physics, underpinned by a discourse of equal rights (producing such inequality as unfair; Balbus 1987; Francis 1999), to explain the under-representation of women in Physics.

Many of the respondents articulating feminist discourses and that of continuing gender discrimination and lack of equality of opportunity in relation to Physics drew on popular social science constructs such as gender stereotyping, gender roles, and socialisation to explain their answers. Allusions to “stereotypes” that men and women are suited to different jobs were especially common: “Again, the stereotype factor, I think that plays a massive part in that [...] That only men do jobs in engineering and stuff like that” (Demi, White English, F, social class 2/3); and “Yeah all those stereotypes and that, that it should be men who do that kind of thing” (Chloe, White English, F, social class 2).

These social science constructs were used to explain how gender-distinct behaviours originate with, and are perpetuated by, society, supporting social rather than gender essentialist accounts for the lack of women in Physics. Joanne (White English, F, social class 2) provides an especially developed illustration of the application of explanatory social science constructs:

J: Well there’s a whole bunch of reasons really that I talked about in my essay sort of—stereotyping, women not being given jobs just because of being women [...] got some stuff about that in there. Toys can sometimes put women off science because … or particularly Physics … because boys’ toys tend to encourage more spatial awareness than girls’ toys do. There’s stereotype threat – I don’t know if you’ve heard of that.

Interviewer: Mm

J: And there’s –

I: What, sorry, “stereotype threat”

J: It’s when you feel that you’re covered by a negative stereotype in a situation where you may exemplify that stereotype, you will put extra pressure on yourself to try and defy it, and in the end choke and just end up exemplifying it in the end anyway. So a lot of women suffer from that, as well “imposter syndrome” where women feel like they’ve got where they are by luck.

Hence in contrast to the elevation of agency and the relegation of structural accounts of inequality to the past produced by equal opportunities/individual choice discourses, the discourses mobilised in these accounts positioned gender dualist and discriminatory practices as ongoing and as impinging on and/or determining women’s behaviour.

The discourse of continued gender discrimination and lack of equality of opportunity in relation to Physics was also expressed within an especially frequent explanation for why women are deterred from pursuing Physics careers—a theme concerning the numerical dominance of Physics by men and the consequent lack of representation of women. It was notable that the concept of “role models” was mentioned by only a few of respondents (three parents and one male student). However the issue of numerical domination by men, as well as the discouraging messages this conveys to women, was an extremely strong theme in our data.

There were a range of different versions of this explanation. Some young women presented the numerical domination by men as itself off-putting and intimidating: “Um … well I mean at the moment it’s mostly boys, so they probably get put off being like ‘Well I’m going to be in a class full of boys, so they don’t want to do it’” (Hannah, White British, social class 1); and “Um … I don’t know, it is definitely like a male job. Like I was looking about [college] like I told you, and my brother was like ‘Well if you’re going to do Physics you will be the only girl in the class’” (Kate, White British, F, social class 1).

Whereas these statements evoke isolation, and almost a fear of obliteration as “the only girl,” others highlighted the potential stigmatisation of being “odd” or “weird” as a minority: “people might you know think a woman doing a, you know a stereotypical man’s job like a builder they might find it strange or something […] that’s probably quite a big factor rather than like a small one, so…” (MacTavish, White, M, social class 4); and

because like in class yeah there’s all the boys who do Physics and like if you’re a girl and you’re good at it, it would be like it just seems a bit weird [...]. I think people just see it as a bit weird. [...] Like I think the boys just like … and because like a lot of boys do it as well, so I think it would just, it’s just like uncomfortable if you’re a woman and you try and go there and I think like the boys in my class they don’t really expect girls to be that smart, so like if you do it [...] you know. (Kelsey, Black Nigerian, F, social class 5)

Kelsey’s words evoke the common association between Physics, masculinity and “cleverness” to which we shall return later in our article. But her words also suggest visceral discomfort and vulnerability at “standing out.”

The lack of representation of women in science was also presented directly as excluding/precluding women’s participation: “Um, yeah there’s been sort of like a stigma with I don’t know. It [women in Physics] doesn’t seem like it’s as prevalent. You don’t see any female scientists being advertised in the same way that like you’ve got male scientists” (Bobster, White, M, social class 3/4); and

But I could imagine that there is still a bit of a kind of “Physics is a man thing.” Cos like for example if you take The Big Bang Theory [a television program] right, like if you think about … obviously well there’s Penny, who’s not even a scientist, but the two girls who are scientists—they’re both biologists. And then all the guys are Physics. So like there is kind of this underlying sort of thing where you’re a bit like “Mm, why is Physics not a girl thing?” Cos Amy Farrar Fowler could easily be a physicist right, and then like you know … for example, I don’t know, like Leonard could probably be a biologist, but they’ve just made it so that all the guys are physicists or engineers, and then the girls … so I guess there is kind of still a little bit of a kind of gender roles sort of deal going on. (Davina, White, F, social class 1)

These statements clearly suggest that the lack of women in Physics (both in actuality and as represented in popular media) sends a message about what is (in)appropriate for women. Here the theme of gender representation overlapped with the afore-mentioned concepts of gender stereotyping in young people’s accounts.

The frequency of references to the TV show “The Big Bang Theory,” especially from young women, to exemplify the gender stereotyping of Physics was highly notable.

um, like have you ever seen Big Bang Theory? [...] It’s four physicists and they’re all men and people don’t really think of that as being sexist, but it is because the female character Penny is like the one that isn’t smart, because she’s a girl and she’s interested in girl things and that’s a whole like running joke on the show, but it’s just sexist. It’s not funny. (Caitlin, White, F, social class 2/3)

The prevalence of the trope of the male scientist in the popular imagination was frequently blamed on the media, and was presented as perpetuating perceptions of science as “for males”: “I don’t really know, it’s probably just … I don’t actually know where my view of that’s come from, but it could be from like movies, the media. Like if you have a mad scientist – it’s always male, it’s never female. It’s just stuff like that I guess” (Poppy, White, F, social class 1); and “I think it’s more because like in the news you’ve got like mainly the people that do are seen as like physicists like Stephen Hawking and people like that. They’re all male overall. You don’t really see many female ones anywhere” (Celina2, White English, F, social class 4). Such statements are supported by research analysis showing the associations between science competence and men/masculinity in the media (e.g., Orthia and Morgain 2016). Indeed, Archer et al. (Archer and DeWitt 2014; Archer et al. 2015) have catalogued in detail the exclusionary power of such imagery and its debilitating impact on the science aspirations of those not inhabiting the projected “appropriate body” (i.e., not male, middle class, and White/South or East Asian).

Comments from Samantha (Indian, F, social class 1) also illustrate how this lack of representation can also be interpreted or conveyed as presenting a “natural” order wherein men are simply better at Physics:

I think in general it [Physics] is perceived as a much more male orientated area of Science. [...] I think I guess in the past women generally didn’t do that much Science. Obviously there are exceptions, but it’s very much like a lot of discoveries have been made by men and I think that’s just carried it on and I think also boys generally tend to have a slightly more kind of “Mathsy,” “Physicsy” brain like a lot of the intelligent boys, so …

Other students critiqued such narratives, maintaining that women’s presence in science has been actively masked:

I don’t see any reason why women should be put off Physics and you know there is no reason, but in a way … like in the same way that people view intelligence as an interest in Science, people view you know Science as all the men at NASA working at their computers with the men landing on the moon and you know people forget that you know there are several prominent women [..]. (Buddy, White English/Italian, M, social class 1)

The importance of representation of women in Physics for “evidencing” the possibility and hence facilitating equity discourses (“possibilising” the notion of the female scientist for respondents) was clear from many responses. For example, Mienie (South Asian, F, social class 2) supports her claim that women can be successful Physicists by highlighting: “because there’s. .. you know there’s a lot of female physicists out there, so—.” Yet equally, it was also noticeable how, rather than necessarily disrupting the monoglossic account of science as a naturally and wholly male terrain, the few heteroglossic cases of women scientists that are publically recognised or included in the curriculum could apparently be easily discounted or ignored, failing to disrupt the monoglossic facade: “Cos like when you see most like discoveries. .. they might just take credit, but like most of them you see them as male. There’s only a few like female scientists like we’ve heard of in science” (Alan, Mixed, White/Black Caribbean, M, social class 3).

Hence our data highlight the importance of representation in terms of “im/possibilising” imagined science futures for young women: their lack of representation in media and ‘real life’ science discursively positions (White and occasionally Asian, middle class) men as ‘naturally’ inhabiting the science terrain, and any women as interlopers, oddities or ‘impossible subjects’ (Butler 1993). In answer to the question as to whether anything deters women from pursuing Physics careers, Tom3 (White, M, social class 1) replies: “Um. .. well I’ve never seen a girl do Physics so I wouldn’t know.” Tom appears to present the lack of young women in Physics as explanatory in itself; a phenomenon that brooks no further speculation. Likewise, for Charlie, gender representation in different subjects naturalises a binary order:

Um, yeah I like I see it as more of a like guy thing. I don’t know why. I just like when I hear Physics I think of like the three nerdy, well I suppose nerdy boys in my year, but they’re really good at it, which is why I think of them, so I don’t really think of girls like because they’re all into beauty and that in my school. (Charlie, White, F, social class 2/3)

Hence, the issue of representation is shown to remain key in young people’s constructions of gender and science and in the im/possibilisation of Physics trajectories for girls and women. The relentless repetition of images that present male bodies in association with Physics, and hence work to embody Physics as male in the public psyche, may be identified and critiqued by respondents, but are nevertheless hard to resist—and impact material practices by constituting and reinforcing Physics as masculine. Our data illustrate the symbolic violence and impact of the repetitive motif of the male Physics body on young women’ perceptions of Physics as an inhospitable, “unnatural,” and potentially isolating route (see also Archer et al. 2016a).

#### The Discourse of Physics as Quintessentially Masculine (and its Supporting Narratives)

As well as being symbolically embodied as male, Physics was also presented by many students as a masculine subject and therefore off-putting and/or inaccessible to girls and women. (As Table 2 shows, this discourse was articulated by the 70 young people on 139 occasions in the data.) As Brittney (White, F, social class 3) explains: “I think because it’s seen as a masculine subject to do and not really feminine [...] It just doesn’t seem very feminine to want to do anything to do with Physics.” There were five distinct narratives supporting this discourse which emerged in response to our question as to whether anything deters women from Physics and Engineering careers: (a) Certain subjects are gender-stereotyped as being masculine or feminine (and hence as appropriate for different genders), (b) Men and women are naturally different and drawn to different subjects, (c) Femininity is antithetical to (masculine) manual work, (d) Femininity is superficial, and (e) Cleverness is masculine, and Physics is a clever/difficult subject.

Although a few students remarked on the construction of Physics as masculine without attributing a reason for this labelling, some drew on the concept of gender stereotyping to position this as an invalid socially perpetuated association. Others drew on the narrative of men and women as naturally different and drawn to different subjects to explain the dearth of women physicists as due to natural phenomena (an account supporting the monoglossic, binarised construction of gender duality):

But I also think that part of it is the way … it’s what interests the male brain as opposed to the female brain. For example computer games, you know we have Xbox in the house and my son plays computer games, and my daughter does rarely because she’s not interested. And so there’s no … it’s not anything to do with sexual discrimination or anything or lack of opportunities. I mean she played girly things … sounds terrible … you know female orientated games when she was younger, but she has no interest in playing computer games now. (Joseph, father of Georgie, White, M, social class 1)

It was interesting to see which subjects were considered appropriate for each gender here. Supporting previous findings, “caring” and “creative” subjects and occupations tended to be seen as appropriate for females (Francis 2002), in comparison to the produced “natural fit” for males with Physics and Engineering (Archer et al. 2012). For example, Hedgehog (White English, M, social class 4) considers that “young women, they’re more into like being like midwives and like beauty therapists and that,” and Cristiano (Black Nigerian, M, social class 5) considers young women “just have other interests,” which he exemplifies as “Probably healthcare.” Additionally, some respondents perpetuated the longstanding construction of Biology as a ‘more feminine’ science discipline: “When I think Physics I think it’s more manly and Biology is more feminine” (Carol, White, F, social class 3).

Biology could be classed a bit. .. not saying it’s more feminine, but women. .. or maybe the females would know more about biology, a) with what we all go through. .. not saying men don’t go through it, but you know we learn a bit more about our bodies and why are bodies are doing things from a lot earlier age with like periods and stuff—we get involved a little bit more about biology earlier on. And we could maybe go more into the medical profession or the caring profession—that side of things. So Biology maybe is a little bit easier or we understand. .. not understand it more, but we can get involved in it easier. (Sally Ann, mother of LemonOnion, White, F, social class 3)

As Sally Ann’s response illustrates, often when probed for rationales for these different gender “dispositions” for different subjects, respondents talked in rather vague terms about different gendered interests in relation to potential subject content in different disciplines. Interestingly, “circuits” were mentioned on numerous occasions as an exemplification of an element of Physics that might deter young women’ interest, either as reported by young women as a topic they didn’t like or as a topic which young men and young women suggested would not appeal to young women. Luna’s (British, F, social class 3) response is indicative here: “Cos I don’t think that electrical circuits really … for a lot of young women might not stand out to them and make them want to do it at A Level … which might be a reason why they don’t continue it.”

Hence, the narratives presented thus far interpolated males and females as different, as well as Physics as more appealing to males, whether due to (spurious) gender socialisation, or to “natural” inherent differences between males and females which render particular subjects more appropriate to one or the other gender. However, the power hierarchy at the heart of the monoglossic, binarised account of gender produced in the discourse of Physics (and Engineering) as masculine emerged more clearly in the three further, inter-related narratives articulated: Femininity is antithetical to (masculine) manual work; femininity as superficial; and cleverness as masculine (and Physics is a clever subject). As we reported previously, many students, especially young men, denied that there was anything deterring women from Physics, but reversed this view in the case of Engineering. For a few, this was about lack of representation. But for the majority, this was due to the association of Engineering with manual work, and manual work with masculinity: “Like isn’t that [Engineering] more manual? So there’d probably be more men in there just for the sake if it being manual labour” (Alan, Mixed, White/Black Caribbean, M, social class 3).

What was intriguing, though, was that none of the accounts positioned physiological differences (e.g., physical strength) as underpinning this envisaged deterrent of manual elements for women. Rather, overwhelmingly, respondents positioned the “problem” as being feminine avoidance of “dirt” or mess: “I was going to say they [women] don’t really like getting dirty and building stuff” (Football Master, White, M, social class 3); and

I don’t have a clear idea on that one, but I think people tend to associate Physics, well no not people, I’m not sure about the general view, but I associate Physics with Engineering and I’ve sort of got a view on Engineering as them building. I see it as a more practical subject with wires and rusty equipment and stuff like that. [...] It’s sort of in the same area in my mind as being a mechanic and stuff like that, so it’s different to Biology which you can see is a very clean and hygienic subject. (Finch, White, M, social class 2/3)

This positioning of femininity as primly “clean” was integrally connected to constructions of femininity as preoccupied with appearance and grooming: “and for a lot of women—they want to wear nice clothes and jewellery, and they just don’t want to wear hard hats” (Naomi, mother of Buddy, White, F, social class 1); “I think that’s why a lot more like men are mechanics and things because it’s hands on. Women don’t want to break their nails do they?” (Louise, White, F, social class 3); and

I do think there’s still a you know a lot of girls that want to do girl things. [...] you’ve still got that type of girl that think they should only do girl things and oh well I don’t want to do that, because that will be, you know they don’t do sport and they don’t do Sciences and they don’t do Maths and stuff like that, because “ooh no what do I want to do that for? I want to go and file my nails and you know do hairdressing and stuff like that.” (Colleen, mother of Caitlin, White, F, social class 2/3) (see also Ghost, Table 2).

What these extracts produce, especially via the vivid misogynist trope of the obsession with “broken nails” and the mimicking of voices, is a denigration of femininity as superficial. As many feminist researchers have recorded, this construction of femininity remains prevalent in educational environments (Francis et al. 2003, Francis 2010; Walkerdine 1989), and is fundamentally intertwined in turn with discourses that produce femininity as dim, vain, inane, and lacking in substance (Other). The counter side to this construction of femininity is of course the animation of the masculine (Subject) as profound, intelligent, reasoning—in other words, the production of intelligence, or cleverness, as masculine. As Walkerdine (1989); Walkerdine 1990) and others have shown, within the gender binary, science and rationality are positioned in association with intelligence and masculinity; the “creative arts” and emotionality, with femininity. “Hard” science is produced both as difficult and as masculine (Archer et al. 2012; Harding 1982; Walkerdine 1989), just as rationality and intelligence are positioned as masculine in the gender binary (Harding 1982, 1989).

The narrative of “Physics as ‘clever’ and masculine” was reported and/or articulated by some respondents, as an explanation as to why fewer women pursue Physics: “I think young men are just thought of to be the smart gender” (Caitlin, White, F, social class 2/3);

Yeah, because there is like some like stereotype things you see that like you can see that oh men are smarter than women, but that’s not always true and like if you want to do, become like a scientist or something like that a woman might be like self conscious and think well I can’t do that, because I will look kind of stupid in front of men, but that’s not always the case. Like women can be smarter than men. (Laura, White, F, social class 2/3); and

well like some young women in my year they act stupid. Like I don’t think they are stupid, but I think they act it. [...] So they think ‘Oh I can’t do it, cos I’m stupid’ – but they’re not at all. *Mm, why do they do that?* Uh … probably because they’re sitting near young men. (Hannah, White, F, social class 1)

Clearly, in each case these young women are reporting what they see as stereotypes, but Hannah’s words especially evoke the ways in which for young women to invest in “masculine/clever” subjects, and/or to actively position themselves as clever, involves a negation of the feminine which may be experienced as untenable (Walkerdine 1990) and indeed as an obliteration of the self (Butler 1993; Walkerdine 1990). As we discuss elsewhere (Archer et al. 2016a), such young women also risk being illegible to, and impossibilised by, others as authentic Physics subjects.

It is worth highlighting that narratives were far from always consistent in participants’ responses, illustrating the jostling, heteroglossic contradiction at play. Perhaps this is unsurprising given the nature of the three key discourses we have identified as operating in discussion of gender and access to Physics: (a) equality of opportunity, (b) continuing gender discrimination in and around Physics, and (c) Physics as quintessentially masculine. Clearly, each of these discourses is in direct tension with the other, hence providing discursive tension and heteroglossic shifts and contradiction within responses when different discourses are drawn upon by respondents to make particular points.

## Discussion

The primary contribution of our findings and analysis has been to identify three key discourses operating within young people’s and their parents’ responses on gender and access to Physics: that of equality of opportunity, that of continuing gender discrimination in and around Physics, and that of Physics as quintessentially masculine. And we have identified how the latter discourse is articulated via five different narratives: (a) Certain subjects are stereotyped as being masculine or feminine (and hence as appropriate for different genders), (b) Men and women are naturally different and drawn to different subjects accordingly, (c) Femininity is antithetical to (masculine) manual work, (d) Femininity as superficial, and (e) Cleverness is masculine and Physics is a clever/difficult subject.

We have illustrated the ways in which these three discourses and five narratives emerged in our respondents’ talk, and we analysed how they positioned gendered subjects and the disciplines of Physics and Engineering in particular ways. Especially, we have shown how the narratives connected to the “opportunities as equal,” meritocratic discourse risk positioning women as responsible for their lack of pursuit of Physics and the continued prevalence of the discourse of Physics as quintessentially masculine. This latter finding supports prior research concerning the longstanding association between masculinity and the Physical Sciences (Archer et al. 2012; Cheryan et al. 2011; Gonsalves 2014; Harding 1991; Walkerdine 1990). Our findings show the resilience of this discourse, and how it continues to permeate the talk of young people and parents, subtly precluding the legitimacy of women’s presence in the physical sciences.

We and other researchers have also illustrated the pervasive association among Science, “intellect” and masculinity (Harding 1982, 1991; Walkerdine 1988, 1990), and the positioning of Physics, especially as a hard subject (the “hardest science”), a masculine subject par excellence (Archer et al. 2016a; Gonsalves 2014). This monoglossic construction is supported by the discourse of physics as quintessentially masculine (and the various narratives expressing this discourse), which, as we have shown, speak to, and perpetuate, the monoglossic, dualistic account of gender, with power invested in the masculine elements of the associated binary (see Francis 2012). We have shown that heteroglossia contests and undermines the hegemony of this account, via both the discourse of gender discrimination in and around Physics and via contradictory utterances reflecting the range of different discourses and narratives at play. Nevertheless, we have also shown that the discourse of Physics as quintessentially masculine retains dominance in its reciprocal support for, and perpetuation by, the monoglossic account of gender. In this way, the lack of representation of women in Physics simply becomes further evidence to support the “naturalness” of men’s domination of Physics. Until these constructions and associations are disrupted, the problem of women’s (lack of) access to Physics, and lower uptake of Physics study at post-16, will clearly continue.

We have also shown the prevalence of a theme concerning women’s (lack of) representation in Physics which was frequently used by respondents to explain women’s lower pursuit of Physics, especially by those articulating the discourse of continuing gender discrimination in and around Physics. We have elaborated the apparent effects of this narrative of women’s lack of representation in perpetuating the construction of Physics as an inhospitable domain for women.

In addition to our discourse analytic approach, our approach was novel in directly asking respondents their views on gender and access to the Physical sciences. Our thematic analysis has shown that young women were more likely than young men to say that women are impeded from accessing Physics and were also more likely to explain this view via an account of gender discrimination and/or social stereotyping. A further finding from our quantitative data supports the existing literature in showing that young women are significantly less likely than are young men to anticipate pursuing the study of Physics after the end of compulsory schooling.

### Limitations and Future Research Directions

Although the research presented here offers several contributions to knowledge, a number of limitations need to be addressed. For example, the results presented in the present study relating to our survey data do not escape the limitations of similar self-report measures (e.g., response bias, control of the sample, spurious responses). However, through conducting repeated in-depth interviews alongside the survey, our work covers both the breadth and depth of participants’ aspirations and constructions of identity, thus reducing any of these threats to validity.

In addition to issues relating to internal validity, several issues regarding the external validity of the research presented in our paper also need to be addressed. Although the results presented can arguably be generalised to secondary school students in England (because our sample was roughly comparable to national figures for Free School Meals eligibility, regional distributions, performance indicators, etc.), wider cultural comparisons need to be made cautiously. Further research replicating these results in other countries would help to build confidence in the generalisability of our findings.

Another issue relating to external validity that should be discussed at this point is the choice of students to be included as respondents to the surveys. Although schools were encouraged to invite a spread of top, middle, and bottom sets, or entire cohorts, to participate, it is possible that teachers self-selected certain classes and students (i.e., top set) to be involved. This likely bias is indicated by the relatively high proportion of students from professional backgrounds included in our sample.

Testing these points, especially generalisability to contexts outside England, comprise possible areas for future research. Additionally, identification and exploration of the specific discourses and narratives underpinning respondents’ discussion of gender and science access provides an important first step in understanding what is necessary to change and confront if access patterns are to alter. However, how this is done, and what strategies might prove most effective in disrupting and challenging these particular narratives, remains unanswered. Hence exploring and trialling such initiatives comprises an important area for future research.

### Practice Implications

Our findings have important implications for those engaged with increasing gender equity, especially those working in the fields of education and/or STEM. There are three main points from our findings that we wish to highlight. The first relates to the positioning of inequality as a thing of the past (via the discourse of opportunities as equal). As we have said, it is important not to disparage young people’s frequent faith in the attainment of equality of opportunity and meritocracy. Nevertheless, they need to be aware of the overwhelming evidence on continued *in*equality in relation to the pursuit of Physics, if to reflect on and confront the remaining binary and discriminatory discursive constructions which we have shown to remain prevalent in their talk. One way to approach this may be for those working with young people to use some of the quotes from the data in the present article to provoke discussion (e.g., in classrooms, workshops and/or youth clubs). This approach of analysing and “deconstructing” illustrative texts on gender construction in reflective group discussion has been shown to be an effective one in both primary and secondary schools (see Davies 1993; Francis 2000), and it might be productively applied to the topic of gender and science access. Moreover, the frequent use of social science concepts by those young people articulating the “continuing gender discrimination in and around Physics” discourse to reject suggestions that opportunities have been equalised and/or that lack of access is the fault of women, suggests that such concepts provide young people with a helpful arsenal to resist and critique other accounts. Hence concepts such as stereotyping, and even discourse analysis, might be elucidated and applied by educators within these discussions (see Davies 1993).

Second, our findings have illustrated the importance of representation, and the negative impact of the lack of women in Physics (both in reality, and as presented in popular media) on perceptions of Physics as a field, and consequently for women’s access to it (and likely for other identities excluded from current representation, e.g., Black and/or working class men). The lack of representation of women in Physics triggers and legitimates a range of narratives, including cyclical assumptions that women’s inability and/or lack of suitability explains their absence. But additionally, our data shows the visceral concerns at minoritisation and/or isolation for young women—whether for being numerically engulfed or for being stigmatised as “weird.” These concerns are not unreasonable, especially when accompanied with a ready store of cautionary tales of discrimination and humiliation that women have endured in entering the Physics and/or engineering fields.

Hence we need to continue work to ensure that women are represented in the Physical sciences: in higher study, in careers, and in educational and media vehicles and materials. Many Physics and STEM associations and Societies are increasingly paying conscious attention to equity in visual representations of the subject. However, it would appear from our findings that these are not yet having sufficient traction or disruptive impact on continued wider representations. Educators and activists continue to have an important role in encouraging media, employers, and educational institutions to recruit and present women in Physics. We need to encourage organisations to take a determined approach to ensuring the appointment and representation of women in key positions and to campaign to persuade the media to adopt a more socially responsible attitude to representation. Findings from Seymour and Hewitt (1997) and others also remind us that recruitment alone is not sufficient: Women and other “non-traditional” STEM students and employees need also to be retained. Campaigns encouraging employers to ensure a supportive environment for women in STEM are productive in this regard, and those of us working in universities may be well-placed to encourage some of the less individualistic pedagogies which Seymour and Hewitt (1997) advocate to retain women and minority ethnic students. (These latter might also help to deconstruct associations between Physical sciences and masculinity.)

This leads to the third issue we wish to highlight: The construction of Physics as a “hard” subject (and thus, as masculine). Some notable exemplar organisations have already acknowledged and embraced this agenda. For example, the UK’s Institute of Physics is leading the way in its attention to gender and its recent agenda to present Physics as an accessible and welcoming subject, rather than a hard one. Drawing on such exemplars, those working with young people in teaching or activist capacities are well-placed to alert young people to the daily relevance of science, as we are demanding accessible and illustrative learning materials that engage rather than deter students. We can also challenge the myth that science is “hard” and difficult, whenever raised by students, colleagues, by our institutional practices or in the media. Only by disrupting the symbolic hegemony which perpetuates the Physical sciences as a masculine and “hard” domain will we increase the presence of women in the sector.

### Conclusion

International research has shown the social and economic benefits of widening participation in STEM. Representation of women has been shown to be especially problematic in the Physical sciences. Having shown that our wider cohort study’s findings support prior research evidence that young women are less likely than are young men to pursue Physics, the present paper explored respondents’ explanations for such patterns. We have drawn on interviews that asked students and parents directly about gender and access to Physics to identify the various discourses and narratives at play in respondents’ talk. These discourses and narratives are frequently contradictory, and a discourse of continuing gender discrimination in the Physical sciences was used by some respondents to contest discourses that position the underrepresentation of women in Physics as “natural” and/or their own fault. However, these latter discourses were very powerful, respectively evoking opportunities as equal or of Physics as quintessentially masculine to produce women as deficient. It is argued that we need to find ways to work with young people to unpack these discourses in order to reflect critically on the status quo. Presentations of STEM as “hard” and difficult need to be challenged to encourage identification and participation from non-traditional STEM students, and diversity of representation in the Physical sciences needs to be taken seriously to the same end. In these ways we may support greater equality of access to the Physical science, benefitting individuals and society.
